# Supplementary material for: A Territory-Wide Study of Arrhythmogenic Right Ventricular Cardiomyopathy Patients from Hong Kong
Source: Rev Cardiovasc Med. 2022 Jun 24;23(7):231. doi: 10.31083/j.rcm2307231 (PMC11266799; doi:10.31083/j.rcm2307231)
Supplement: Supplementary file 1 [file 2153-8174-23-7-231-s1.doc]

**Supplementary Table 1. Cox proportional-hazards model to predict incident VT/VF.**

|  | Incident VT/VF | |
| --- | --- | --- |
|  | Univariate Hazards Ratio  (*p* value; 95% CI) | Multivariate Hazards Ratio  (*p* value; 95% CI) |
| Female gender | 0.66 (0.36-1.19; *p* = 0.170) |  |
| Family history of ARVC/D | 0.49 (0.12-2.03; *p* = 0.328) |  |
| Family history of VF/SCD | 1.16 (0.79-2.49; *p* = 0.737) |  |
| Pre-existing VT/VF | 1.44 (0.81-2.55; *p* = 0.210) |  |
| Syncope | **1.83 (1.04-3.23; *p* = 0.036)** | 1.27 (0.68-2.37; *p* = 0.76) |
| Palpitations | 1.77 (0.97-3.22; *p* = 0.065) |  |
| LVEF | 0.98 (0.96-1.00; *p* = 0.117) |  |
| QRS duration | **1.02 (1.008-1.03; *p* < 0.001)** | **1.01 (1.0002-1.02; *p* = 0.013)** |
| QTc duration | 1.00 (0.99-1.01; *p*=0.330) |  |
| PR interval | 1.00 (0.99-1.003; *p* = 0.400) |  |
| R wave amplitude in V5 | 0.63 (0.34-1.15; *p* = 0.169) |  |
| S wave amplitude in V1 | 0.79 (0.32-1.99; *p* = 0.719) |  |
| Epsilon waves | **2.34 (1.22-4.48; *p* = 0.011)** | 1.46 (0.83-3.64; *p* = 0.144) |
| Premature ventricular contractions | 1.18 (0.65-2.12; *p* = 0.583) |  |
| TWI in any lead except aVR/V1 | 1.53 (0.81-2.92; *p* = 0.188) |  |
| TWI in 2/3 inferior leads | 1.77 (0.984- 4.48; *p* = 0.057) |  |

*Abbreviations: VT/VF: ventricular tachycardia/ventricular fibrillation; VF/SCD: ventricular fibrillation/sudden cardiac death; LVEF: left ventricular ejection fraction; TWI: T-wave inversion

**Supplementary Table 2. Cox proportional-hazards model to predict new-onset HFrEF.**

|  | New-onset HFrEF | |
| --- | --- | --- |
|  | Univariate Hazards Ratio  (*p* value; 95% CI) | Multivariate Hazards Ratio  (*p* value; 95% CI) |
| Female gender | **2.72 (1.13-6.54; *p* = 0.025)** | **4.72 (1.85-12.02; *p* = 0.001)** |
| Family history of ARVC/D | N/A |  |
| Family history of VF/SCD | 0.80 (0.18-3.42; *p* = 0.764) |  |
| Pre-existing VT/VF | 0.89 (0.37-2.18; *p* = 0.810) |  |
| Syncope | 0.96 (0.41-2.26; *p* = 0.931) |  |
| Palpitations | 1.06 (0.46-2.42; *p* = 0.895) |  |
| QRS | 1.01 (0.99-1.02; *p* = 0.086) |  |
| QTc duration | **1.01 (1.008-1.02; *p* < 0.001)** | **1.02 (1.01-1.03; *p* = 0.004)** |
| PR interval | 1.00 (0.99-1.01; *p* = 0.811) |  |
| R wave amplitude in V5 | 0.69 (0.32-1.49; *p* = 0.351) |  |
| S wave amplitude in V1 | 0.15 (0.02-1.19; *p*=0.073) |  |
| Epsilon waves | **4.29 (1.85-9.95; *p* = 0.001)** | **2.86 (1.21- 6.76; *p* = 0.017)** |
| Premature ventricular contraction | 2.40 (0.88-6.50; *p* = 0.088) |  |
| TWI in any lead except aVR/V1 | **12.8 (1.72-95.73; *p* = 0.013)** | **8.36 (1.07-65.3; *p* = 0.043)** |
| TWI in 2/3 inferior leads | 1.92 (0.84-4.38; *p* = 0.121) |  |

**Supplementary Table 3. Cox proportional-hazards model to predict all-cause mortality.**

|  | All-cause mortality | |
| --- | --- | --- |
|  | Univariate Hazards Ratio  (*p* value; 95% CI) | Multivariate Hazards Ratio  (*p* value; 95% CI) |
| Female gender | **3.45 (1.11-10.76; *p* = 0.030)** | 2.51 (0.54-11.7; *p* = 0.242) |
| Family history of ARVC/D | N/A |  |
| Family history of VF/SCD | 1.31 (0.29-5.90; *p* = 0.719) |  |
| Pre-existing VT/VF | 0.63 (0.19-2.06; *p* = 0.449) |  |
| Syncope | 1.39 (0.49-3.97; *p* = 0.531) |  |
| Palpitations | 1.73 (0.50-5.28; *p* = 0.338) |  |
| LVEF | **0.93 (0.89-0.97; *p* = 0.001)** | **0.89 (0.81-0.97; *p* = 0.008)** |
| QRS | **1.03 (1.01-1.04; *p* = 0.001)** | **1.04 (1.01-1.78; *p* = 0.01)** |
| QTc duration | **1.01 (1.001-1.03; *p* = 0.029)** | 0.99 (0.98-1.02; *p* = 0.639) |
| PR interval | 0.99 (0.98-1.00; *p* = 0.055) |  |
| R wave amplitude in V5 | 0.92 (0.42-2.02; *p* = 0.834) |  |
| S wave amplitude in V1 | 0.09 (0.006-1.46; *p* = 0.091) |  |
| Epsilon waves | **4.21 (1.45-12.19; *p* = 0.008)** | **6.33 (1.16-34.31; *p* = 0.033)** |
| PVC | 1.93 (0.59-6.37; *p* = 0.279) |  |
| TWI in any lead except aVR/V1 | 1.53 (0.48-4.91; *p* = 0.472) |  |
| TWI in 2/3 inferior leads | 1.75 (0.60-5.00; *p* = 0.304) |  |

**Supplementary Table 4a. Scoring system for VT/VF.**

| **Variable** | **Univariate Hazards Ratio (95% CI)** | ***p*-value** | **Points** |
| --- | --- | --- | --- |
| Epsilon wave | 2.34 (1.22-4.48; *p* = 0.011) |  | 1 |
| Syncope | 1.83 (1.04-3.23; *p* = 0.036) |  | 1 |
| QRS > 98.5 | 2.87 (1.53-5.43; *p* = 0.001) |  | 1 |

**Supplementary Table 4b. VT/VF score characteristics of patients with/without incident VT/VF.**

|  | **With VT/VF** | **Without VT/VF** | ***p* value** |
| --- | --- | --- | --- |
| Median risk score for VT/VF (IQR) | 1.0(1.0-2.0) | 0.0(0.0-1.0) | <0.0001 |

**Supplementary Table 4c. Stratification performance of VT/VF score**

|  | **HR (95% CI)** | **Z value** | ***p*-value** |
| --- | --- | --- | --- |
| VT/VF score (per unit) | 1.74 (1.30-2.33) | 3.76 | <0.0001 |
| VT/VF score 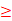 1 | 2.08 (1.17-3.7) | 2.49 | 0.013 |

*determined by maximal rank statistics approach (Figure 2, left panel)

**Supplementary** Table 5a. Scoring system for new-onset HFrEF

| **Variable** | **Univariate Hazards Ratio (95% CI)** | ***p*-value** | **Points** |
| --- | --- | --- | --- |
| Female Gender | 2.72 (1.13-6.54) | 0.025 | 1 |
| Epsilon wave | 4.29 (1.85-9.95) | 0.001 | 2 |
| TWI in any lead except aVR/V1 | 12.8 (1.72-95.73) | 0.013 | 6 |
| QTc > 437.5ms | 9.81 (2.90-33.1) | 0.001 | 5 |

**Supplementary Table 5b. HFrEF score characteristics of patients with/without HFrEF**

|  | **Patients with HFrEF** | **Patients without HFrEF** | ***p* value** |
| --- | --- | --- | --- |
| Median risk score for HFrEF (IQR) | 12 (11-13) | 6 (1-7) | <0.0001 |

**Supplementary Table 5c. Stratification performance of HFrEF score**

|  | **HR (95% CI)** | **Z value** | ***p*-value** |
| --- | --- | --- | --- |
| HFrEF score (per unit) | 1.48 (1.26-1.74) | 4.70 | <0.0001 |
| HFrEF score 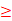11 | 15.91(5.37-47.17) | 4.99 | <0.0001 |

*determined by maximal rank statistics approach (Figure 2, right panel)

**Supplementary** Table 6a. Scoring system for all-cause mortality

| **Variable** | **Univariate Hazards Ratio (95% CI)** | ***p*-value** | **Points** |
| --- | --- | --- | --- |
| Female Gender | 3.54 (1.11-10.76) | 0.030 | 2 |
| Epsilon wave | 4.21 (1.45-12.19) | 0.008 | 2 |
| QRS > 122.5 | 6.77 (2.34-19.57) | < 0.001 | 3 |
| QTc > 448.5 | 6.05 (1.68-21.8) | 0.006 | 3 |

**Supplementary Table 6b. Mortality score characteristics of patients with/without all-cause mortality**

|  | **Mortality** | **Alive** | ***p* value** |
| --- | --- | --- | --- |
| Median risk score for mortality (IQR) | 6.5 (5-8) | 2 (0-4) | <0.0001 |

**Supplementary Table 6c. Stratification performance of mortality score**

|  | **HR (95% CI)** | **Z value** | ***p*-value** |
| --- | --- | --- | --- |
| Mortality score (per unit) | 1.65 (1.33-2.06) | 4.51 | <0.0001 |
| Mortality score 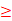3 | 22.59 (4.94-103.3) | 4.02 | <0.0001 |

*determined by maximal rank statistics approach (Figure 2, bottom panel)
